# Impact assessment of particulate pollution on maternal mortality in Nigeria

**DOI:** 10.1038/s41598-022-19518-5

**Published:** 2022-11-16

**Authors:** M. E. Emetere, T. E. Oladimeji

**Affiliations:** 1 Physics Department, Bowen University, Iwo, Osun State Nigeria; 2 Department of Mechanical Engineering, University of Johannesburg, Johannesburg, South Africa; 3 Chemical Engineering, Covenant University, Ota, Ogun State Nigeria

**Keywords:** Environmental sciences, Natural hazards, Risk factors, Physics

## Abstract

Recently, the World Health Organization reported that 20% of all global maternal deaths happened in Nigeria between 2005 and 2015. In developing countries, these maternal deaths are mainly from air pollution. Due to poor facilities and documentation, the extent of danger is not known. This research seeks to estimate the available pollutants and its direct and indirect impact on maternal mortality. Ten (10) years (2010–2019) datasets of black carbon, sulfur dioxide, dust, carbon monoxide, organic carbon particulates, sea-salts, and sulphate particulates were obtained from the second modern-era retrospective analysis for research and applications (MERRA-2). The dataset was obtained for the six geopolitical zones of Nigeria and analyzed using statistical tool, models, spatial interpolation, and risk analysis. The volumetric and radioecological risk was also analyzed. It was observed the dust content had minute volume of heavy metal and/or radionuclide particles that may be unharmful in the short term but lethal in the long term. The risk quotient and total dose rate per organism are given as 0.00000396 and 0.0000396 µGy h^−1^. The result in this manuscript corroborates existing data on maternal mortality in Nigeria. It is recommended that the safety of pregnant woman depends on significant efforts of authorities to enact and enforce environmental laws to mitigate air pollution.

## Introduction

Air pollution releases toxic or harmful particles and gases known as pollutants through various means such as fumes from industries, burning bushes, emissions from automobiles, dust from natural emissions, mold spores, and lots more. Due to various forms of urbanization and industrialization, many harmful substances have been released into the atmosphere, affecting air quality in various geographical areas/regions. These toxic or harmful pollutants include different levels of particulate matter (PM), carbon monoxide (CO), oxides of sulphur (SO_x_), oxides of nitrogen (NO_x_), hydrocarbons^1^, to mention but a few. Dangers of prolonged exposure to air pollution to man and environment could result in coughing, eye irritation, chronic fatigue, asthma, bronchitis, cancer, lung or heart damage, and ultimately death. Other impacts of air pollution include the death of wildlife, acid rain, global warming, eutrophication, depletion of the ozone, and climate^2^.

Research has shown  over the years that ambient air pollution affects human health. Previous works have shown that air pollution is significant in adult or children's mortality and affects the environment at large^1,3,4^. As pollution grew year in and year out, various discoveries on methods and materials could be used to reduce the effect of pollution such as substituting coal for gas for heating, tree planting and the use of eco-friendly materials^5^. Other solutions to air pollution include; reducing fossil fuels burned, reuse and recycling, clean energy  adaption, and usage of energy-efficient devices.

History of air pollution can be traced from combustion processes, to high rise in industries and urbanization. An increase in cars and other means of transportation is another primary source which has added to world pollution. Air pollution could occur due to human activities, anthropogenic or through nature, biogenic e,g volcanic eruption. Air pollution poses a public global health threat to people of all age groups, but pregnant women are more susceptible than others^6,7^. Since the fetus receives oxygen from the mother, breathing polluted air affects the fetus, and over-exposure of pregnant women to the polluted air at an early stage of pregnancy can result to premature birth. Fetuses are highly susceptible to pollutants due to their physiological immaturity^8^.

In this research, the impact of air pollutants on maternal deaths in Nigeria was examined using primary and secondary dataset that elucidates urgent government intervention in curbing outdoor and indoor pollution. The research hypothesis of this study is hinged on World Health Organization (WHO) report that 20% of global maternal death is from Nigeria. Based on this hypothesis, the air pollutants was investigated based on the six geo-political regions in the country.

### Air pollution impacts on maternal health

 Recent reports show that air pollution is associated with increased risk of adverse pregnancy outcomes, affecting both the mother and the fetus^6^. The developing fetus solely depends on the mother for survival and could be adversely affected when the mother is at risk. A large number of research had admitted that air pollution is recognized as one of the risk factors for numerous diseases in pregnant women^9–11^. Overexposure of pregnant women (PG) to polluted air could put the fetus's life in danger and result in growth retardation, low birth weight, preterm delivery, and childhood mortality^3^. Rochas et al.^12^ reported that reducing birth weight and the placenta could be traceable to outdoor air pollution. Preterm delivery in cases of babies born before the due time; there have also been shreds of evidence of association of air pollution at preterm birth, as concluded by Ref^13–15^. Hypertensive disorder can be caused by air pollution derived from particulate matter (PM_10_ and PM_2.5_), CO, sulphur(IV)oxide (SO_2_), and ozone (O_3_). The preliminary ways pregnant women can protect themselves from polluted air include: get an air purifier, grow trees all around, and reduce indoor pollution. However, the World Health Organization (WHO) has set some air quality guidelines (AQG), which serve as a preventive measure to pollutants' emissions and give a permissible pollutant concentration at a particular averaging period^16^. As stated by Malmqvist et al.^17^, current AQG may not cover or protect pregnant women to the full extent, which implies that pregnant women are still at significant risk of air pollution.

Every pregnancy stage comes with various changes that affect the body systems, starting from increased body weight to change in appetite and hormonal and immune system changes. All these changes can make a pregnant woman more vulnerable to diseases and infections, resulting in complications in severe cases^18^. Change in the immune system is necessary during pregnancy to protect the mother and her fetus. For mother and baby safety, some immune or hormones must be enhanced while others are suppressed^18^. Each trimester has its associated challenges that differ for the individual; some other hormones include reducing lung capacity, frequent urination, blood flow changes that modulate immune responses, to mention but few. Furthermore, most severe cases are noticed in the third-trimester stage^19^.

Also, hormonal change can increase infection risk in pregnant women, especially on the urinary tract (UT). This effect on the tract is tremendous as it leads to UT infection due to increase secretion of progesterone hormone, which causes the bladder muscles and ureter to relax, thereby leading to prolonged urine in the bladder^20^. In some cases, change in hormones makes some pregnant women susceptible to yeast infection called candidiasis; this is produced due to high estrogen levels in the reproductive organ^21^.

Also, an increase in the lungs' fluid level may lead to susceptibility to pneumonia, a lung infection. The size of the foetus exerts more pressure on the abdomen and lungs due to increased fluid in the lungs, which may eventually result in a buildup in the lungs and stimulate bacterial growth in the body, thereby reducing body resistance disease^22^.

### Maternal mortality in Nigeria

The maternal mortality ratio is the number of women who die from pregnancy-related causes. Maternal mortality in Nigeria is common in women during childbearing age, i.e., pregnant women. It was reported that over 600,000 maternal deaths occurred between 2005 and 2015 in Nigeria^23^. Most of the deaths are traceable to hypertensive disorders of pregnancy, postpartum hemorrhage, and unsafe abortion. Meh et al.^24^ examined maternal mortality in north and south of Nigeria. They observed more maternal deaths in the north of the country than in the southern region. The deaths were traceable to the low health services in the north as well as the poverty levels. Also, insurgency and literacy are other factors that were considered to increase maternal deaths in Nigeria. Sageer et al.^25^ reported that the average age of maternal death in parts of southern Nigeria was 30.8 ± 5.7 years.

**Figure 1:**
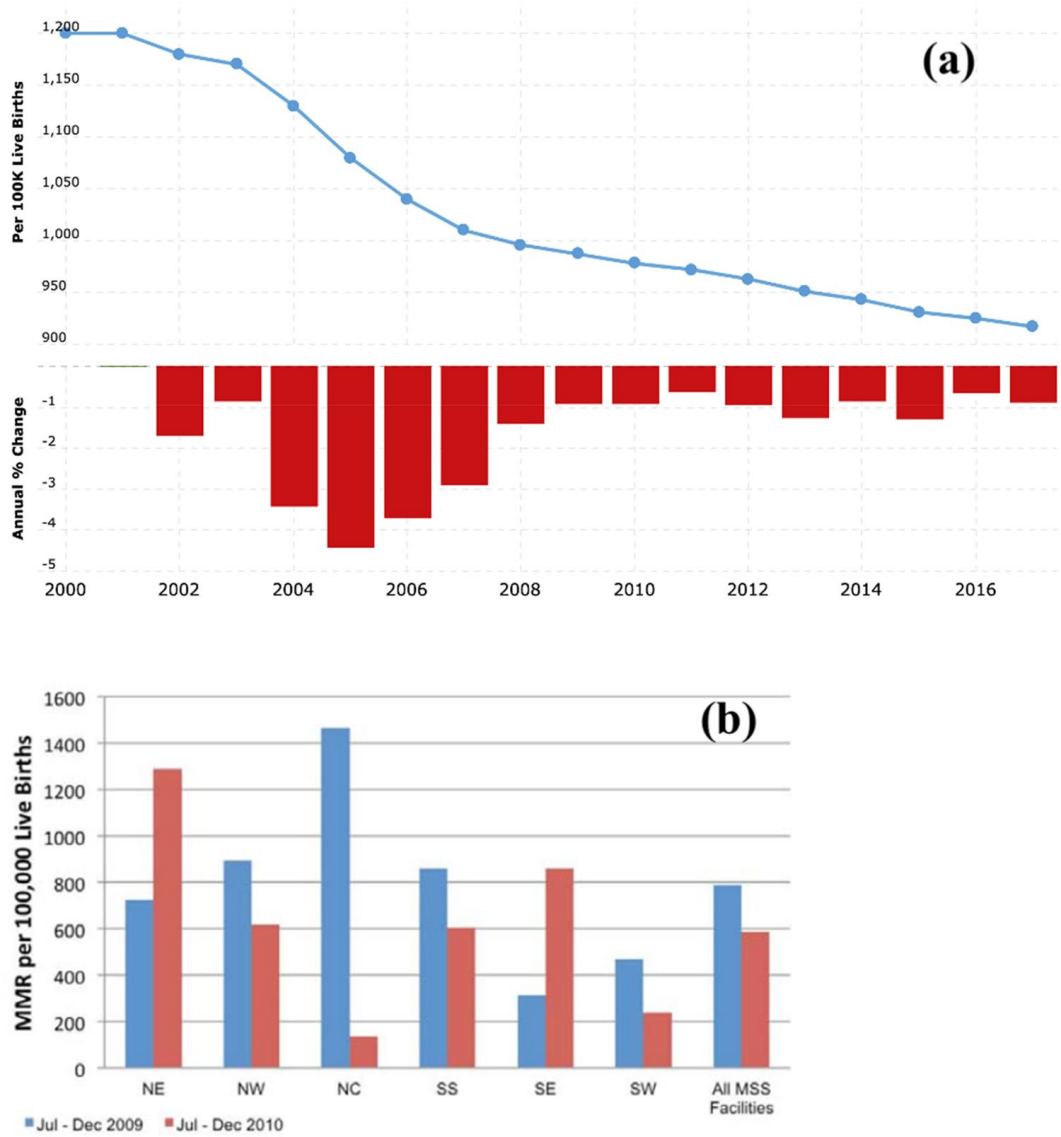
(**a**) Maternal mortality rate in Nigeria^26^ (**b**) Maternal mortality ratios for different geopolitical zones in Nigeria^28^.

World Bank report shows that the maternal mortality ratio is 917 per 100,000 live birth). More specifically, it was reported that the number of women who die from pregnancy-related diseases or pollution are within 42 days of pregnancy ^26^. Figure 1a shows that the mortality rate keeps decreasing yearly from 2.47% to 0.64% due to improved lives and increased public health centers to about 34,000^27^. However, Fig. 1b shows that maternal mortality is highest in the north-central (NC) geopolitical zones than other regions (north-east (NE), north-west (NW), south-west (SW), south-south (SS), and south-east (SE)).

Among the salient points in the Sustainable Development Goals (SDG) is the reduction of global maternal mortality to less than 79 per 100,000 live births by 2020. In low-income countries, access to skilled health-personnel during pregnancy is critical. Air pollution can be human-made or natural. While known techniques can control human-made induced air pollution, natural air pollution sources are beyond man's control. For example, the Sahara Desert has proximity to Nigeria; hence, the influx of Sahara dust is a common contamination source. Unfortunately, there are no data to trace the history of pregnancy in Nigeria. In this study, the focus is to estimate the influence of natural source of pollution on pregnant women.

### Observed air pollutants in Nigeria

**Figure 2:**
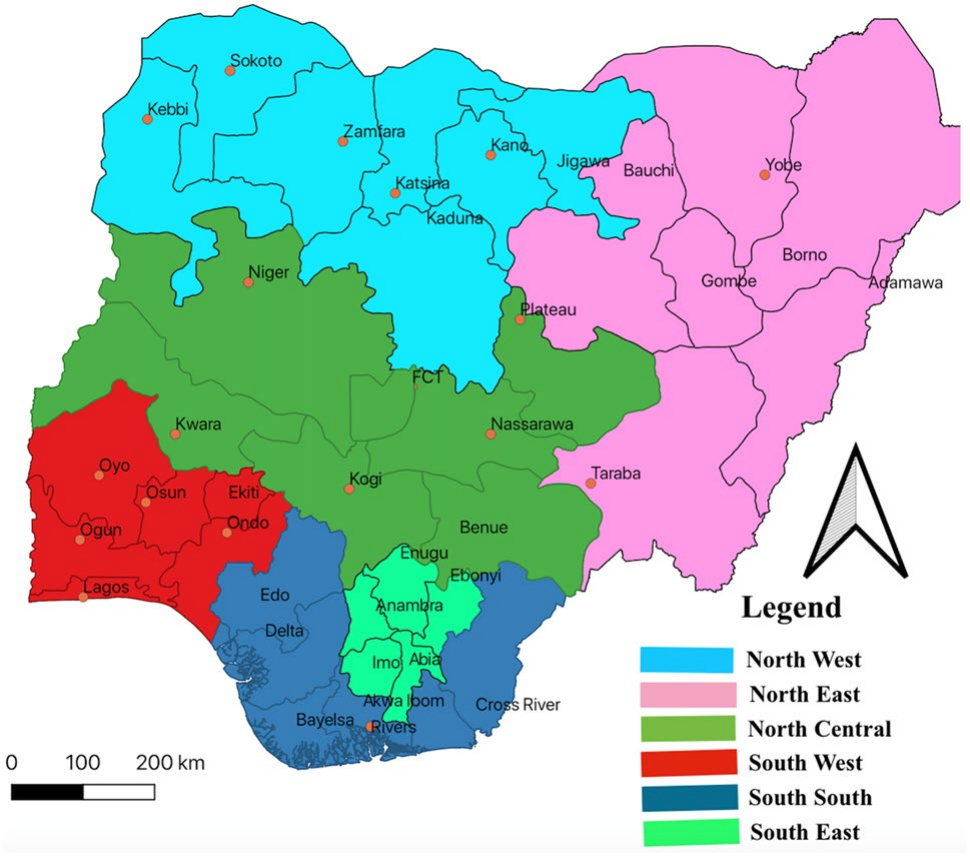
Nigeria geopolitical zones.

Nigeria has six geopolitical zones (GZ), as presented in Fig. 2. Figure 2 was produced using Quantum Geographical Information System (QGIS) software^29^. The female population in Nigeria is about 107,486,776^30^ in which 64% are in their reproductive age. The type of anthropogenic emission over different geopolitical zones is peculiar to its vegetation, human population, and infrastructure. For example, in the south-south geopolitical zone, gas flaring is the major among bush burning, automobile emission, fumes from illegal refineries, etc.; in the south-west geopolitical zone, industrial emission is the major among bush burning, automobile emission, biomass burning, etc.; in the south-east geopolitical zone, automobile emission is the major among bush burning, industrial emission, biomass burning, etc.; in the north-central geopolitical zone, biomass burning is the major among automobile, industrial pollution, Sahara dust influx, etc.; in the north-west and north-east geopolitical zones, Sahara dust influx is the major among biomass burning, automobile emission, bush burning, etc.^31–34^.

One of the prominent pollutants is particulate matter (PM). This pollutant is a class of physically and chemically discrete particles of different sizes. It may be from anthropogenic or natural sources. Dust particles are often referred to as particulate matter (PM). Particles could be emitted directly or formed from the transformation of gaseous pollutants such as VOCs, SO_x_, and NO_x_. Most time, it is in the form of dust particulates. Particles with diameter of less than 10 µm (PM_10_ are more dangerous and travel faster than those above 10 µm. These small particles quickly enter the nose, trachea, and lungs and result in serious respiratory problems^35^. Some research has linked PM exposure to high risk of infant mortality and low birth weight^11,36^. To minimize this pollutant's effect, each state's air quality index (AQI) should be available and accessible to determine the actual PM level permitted in that locality^37^. Another prominent pollutant is carbon monoxide (CO). This pollutant is known to be a silent killer because it is toxic, odourless and colourless gas^35^. Incomplete combustion, which results from the burning of fuel in industries, generators in enclosed space, unvented gas, and cigarette smoke are some of the CO emissions sources. This gas is one of the most harmful gas for a pregnant woman as it may reduce the oxygen in the mother's blood leading to failure in delivering oxygen to the fetus; exposure at an early stage of pregnancy could also lead to preterm birth, cardiac birth defect and retarded growth^38^. Some of the preventive measures include installing CO alarm in industries or homes to alert people of CO emissions^39^. Mkpe et al.^40^ analyzed the sources of CO pollution in Nigeria as presented in Table 1.

**Table 1:** Sources of CO pollution in Nigeria^40^.

Source of exposure	Ambient or indoor air concentration of CO	Life-stage of exposure
Domestic sources-generators firewood and kerosene	70 kt/year	General population
Road traffic	Lagos = 10–250 ppm PH = 5.0–61.0 ppm Warri = 1.0–52 ppm	General population
Nigeria is the leading gas flaring country in the world	Around 24 billion cubic meters of gas (45%) of its total gas production) flared in 2004	Boasts ambient CO air in the Niger Delta
Gas flaring from Nigerian refineries	Tons of CO/year Kaduna-56,738.56, Port Harcourt phase 1-12,973.20, Port Harcourt phase 2-104,230.14, Warri-67,117.96	General population
Gas flaring	2.49 Tg/yr CO representing 12% of total CO emissions in Nigeria in 1995	General population
Smoking vehicles on Nigeria delta roads	Ambient CO µg/m^3^ Mbiama = 191; > 150 in Bonny, Brass, Nchia Port Harcourt, Sapele and Ughele; > 100 in Omoku, Owasa, Eket, Buguma, Ahoada and 100 µg/m^3^ in Warri and Odukpani Emuohia and Ukwugba- > 50 µg/m^3^	General population including market women

**Figure 3:**
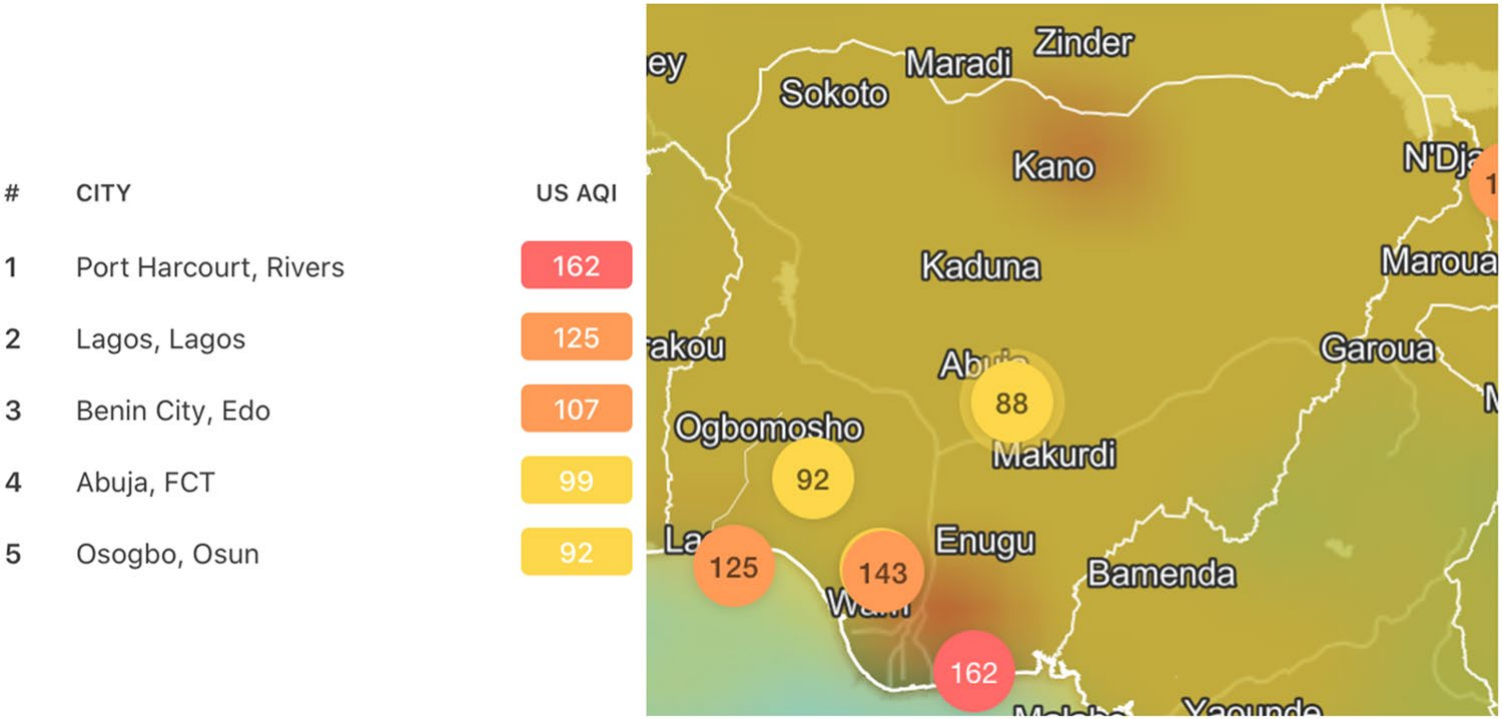
Air quality over selected cities in Nigeria^43^.

Aside CO, there are other dangerous pollutants that occur in Nigeria as anthropogenic pollution. Sulphur IV oxide (SO_2_) is a significant pollutant wherever there is automobile or industrial emission. The primary source of SO_2_ is from the burning of fossil fuels, coal, and natural gas. Other sources include electric power generating plants, transportation, energy production, and combustions, to mention but few^41^. Its effect includes irritations, lung damage, acid rain. Hou et al.^42^ conducted a case study on the relationship between SO_2_ and stillbirth feral death and concluded that pregnant women exposed to this pollutant in their first trimester have fetal death. Nitrogen IV oxide (NO_2_) is another major pollutants wherever there is an automobile or industrial emission. Road transport automobiles is the largest source of nitrogen emissions. Other sources include production and combustion processes, fossil fuel extraction, etc. it could result in the accumulation of excess fluid in the lungs called pulmonary edema. Findings from Iñiguez et al. (2016) suggest prenatal exposure to NO_2_ affects the development of a fetus, reducing its length and head circumference. IQAir^43^ gave the air quality over major cities as indicated in the figure below (Fig. 3). It can be seen that the distribution of air pollutants in Nigeria is peculiar to locations. Oil producing locations and large cities are more polluted with significant concentrations of CO, NO_2_, SO_2_, etc.

## Methodology

### Data generation

The raw dataset of dust deposition, dust column u-wind mass flux, and dust column v-wind mass flux were sourced from the Modern Era Retrospective-Analysis for Research and Applications (MERRA-2). MERRA dataset is sourced from NASA’s Earth Observing System satellites. The information used was 10 years (2010–2019) dataset over Nigeria. The spatial grid of the dataset was 3D, model-level, full horizontal resolution. The dataset was obtained yearly. U-wind is the zonal velocity and it is the component of the horizontal wind towards the east. V-wind is the meridional velocity and it is the component of the horizontal wind towards the north. The dust column u-wind mass flux is the influence of the zonal velocity in the dust column distribution, while the dust column V-wind mass flux is the influence of the meridional velocity in the dust column distribution.

The second data exploration was done via sampling each location from each geopolitical zones of the country. The geopolitical zones have been displayed in Fig. 2. The locations were Lagos (south-west GZ), Aba (south-east GZ), Abuja (north-central GZ), Kano (north-central, Port Harcourt (south-south GZ), and Borno (north-east GZ). These locations are the most polluted location within each geopolitical zones^31–34,44,45^. The spatial grid of the dataset was 2D, single-level, full horizontal resolution. The dataset was obtained monthly for 10 years between 2010 and 2019.

### Data analysis

The raw dataset was treated using the statistical technique. The outliers were referred to as noise. The noise was deleted and replaced using an artificial neural network (ANN). After treating the dataset, several statistical operations were performed. The first statistical operation was finding the percentile of the dataset using Eq. (1).

1 $${\text{PR}} = \left[ {\frac{{{\text{CF}} + 0.5F}}{N}} \right] \times 100$$

where PR is percentage rank, CF is the cumulative frequency below the given score, F is the frequency of the given score, and n is the number of scores in the distribution. The upper and lower limit is estimated using Eqs. (2).

2 $${\text{Rank }}\left( {\text{Upper Limit }} \right) \, = \, \left( {{1}00 \, - {\text{ percentile}}} \right) \, * \, \left( {\text{ Total number of dataset}} \right) \, /{ 1}0000$$

3 $${\text{Rank }}\left( {\text{Lower Limit }} \right) \, = \, \left( {{99}.{99 } - {\text{ percentile}}} \right) \, * \, \left( {\text{ Total number of dataset}} \right) \, /{ 1}0000$$

The basic statistical calculation that borders on standard deviation, standard error, and variance were calculated. The correlations of the dataset were performed using the Pearson and Spearman correlation. The Pearson correlation was calculated using Eq. (4), while the Spearman correlation was estimated using Eq. (5).

4 $$\rho_{X,Y} = \frac{{Cov\left( {X,Y} \right)}}{{\sigma_{X} \sigma_{Y} }}$$

where cov is the covariance, $\sigma_{X}$ is the standard deviation of X, and $\sigma_{Y}$ is the standard deviation of Y.

5 $$\rho = 1 - \frac{{6\sum d_{i}^{2} }}{{n\left( {n^{2} - 1)} \right)}}$$

where *ρ* is the Spearman rank correlation, di is the difference between the ranks of corresponding variables, and n is the number of observations.

The spatial interpolation was done using the quantum geographic information system (QGIS). It is free and open source used to plot Fig. 5. The imagery from the MERRA was modified using MATLAB. This technique was used to plot Figs. 6,7 and 8.

### Mathematical experimentation and risk assessment

The risk analysis was performed using mathematical experimentation. The second-order differential equation of particulate transport through human airways was given by Shunshoku et al. (2006):

6 $$P_{ao} \left( t \right) + a_{1} \dot{P}_{ao} \left( t \right) = f_{E} \left( V \right)V\left( t \right) + g_{R} \left( V \right)\dot{V}\left( t \right) + b_{2} \ddot{V}\left( t \right) + P_{eea} + \varepsilon \left( t \right)$$

where $P_{ao} \left( t \right)$ Is the airway opening pressure, $P_{eea}$ is the end-expiratory alveolar pressure. $\varepsilon \left( t \right)$ contains the modeling error and the measurement noise, $f_{E} \left( V \right)$ is the pulmonary elastance, $g_{R}$ is the airway resistance, $V\left( t \right)$ is the air-volume of the lung.

When there is voluntary or involuntary movement around the oropharynx, the lung volume is said to be chaotic because of vibrations along the lining of the lungs. In this case, $\dot{P}_{ao} \left( t \right) = 0$. Then, Eq. (6) would no longer be calculated in the lungs' air-volume but the particulates' radius. Hence, Eq. (6) becomes

7 $$\frac{1}{\varepsilon \left( t \right)}\left[ {b_{2} \ddot{r}\left( t \right) + g_{R} \left( r \right)\dot{r}\left( t \right) + f_{E} \left( r \right)r\left( t \right) = s} \right]$$

where $s = P_{eea} - P_{ao} \left( t \right){\text{s}} = {\text{f}}_{{\text{E}}} \left( {\text{V}} \right){\text{V}}\left( {\text{t}} \right) + {\text{P}}_{{{\text{eea}}}} + {\upvarepsilon }\left( {\text{t}} \right) - {\text{P}}_{{{\text{ao}}}} \left( {\text{t}} \right)$ the initial conditions and $\dot{r}\left( 0 \right) = 0.0393$. Therefore, the aerosols that would be entering the human body is described by Eq. 8 when *b*_2_ = 5, *g*_R_ = 1, *f*_E_ = 2, *s* = 0.5.

The risk assessment was calculated using USEPA exposure factors handbook^37^:

8 $$ADD = \frac{C \times InhR \times EF \times ED}{{Bw \times AT}}$$

where ADD is average daily dose (μg/kg/day), *C* is the mean dust Concentration, InhR is the inhaled rate (1 mg/day for adults, 2.5 mg/day for pregnant women and 2 mg/day for children), EF is the exposure frequency of 365 days per year, ED is 6 years for children and 24 years for adults, Bw is the bodyweight of 60 kg for adults and 15 kg for children was assumed, AT is the averaging time of 1300 days in 5 years.

The radiological risk analysis from the inhaled radioactive particulates in dust was estimated using the ERICA 1.3 software. The radioecology modalities are already reported in Ref^46^.

The dose rates are calculated using the equivalent dose for radiation Λ (which can be α, β, γ, X, n, etc.) using

9 $$H_{\Omega } = \mathop \sum \limits_{ \wedge } w_{ \wedge } D_{\Omega \Lambda }$$

where w_Λ_ is the radiation weighting factor, D_ΩΛ_ is the absorbed radiation, and it is measured in Gy w_Λ_ allows the introduction of Sv as the unit for H_Ω_. The total equivalent dose for the whole organism is therefore calculated as:

10 $$H = \mathop \sum \limits_{{\Omega }} w_{{\Omega }} H_{{\Omega }}$$

Inskip et al.^47^ gave the internal dose rates from lead and its isotope in bone to blood of nonhuman primate as presented in Table 2.

**Table 2:** Lead and its isotope influence in the human body^47^.

Dose solution	Abundance of ^204^Pb (%)	Abundance of ^206^Pb (%)	Abundance of ^207^Pb (%)	Abundance of ^208^Pb (%)
Common lead	1.4	24.8	21.7	52.1
^204^Pb-enriched	17.9	22.5	18.1	41.5
^206^Pb-enriched	0.98	45.4	17.1	36.5
^207^Pb-enriched	1.1	19.5	38.3	41.1

### Discussion on general pollution state

**Figure 4:**
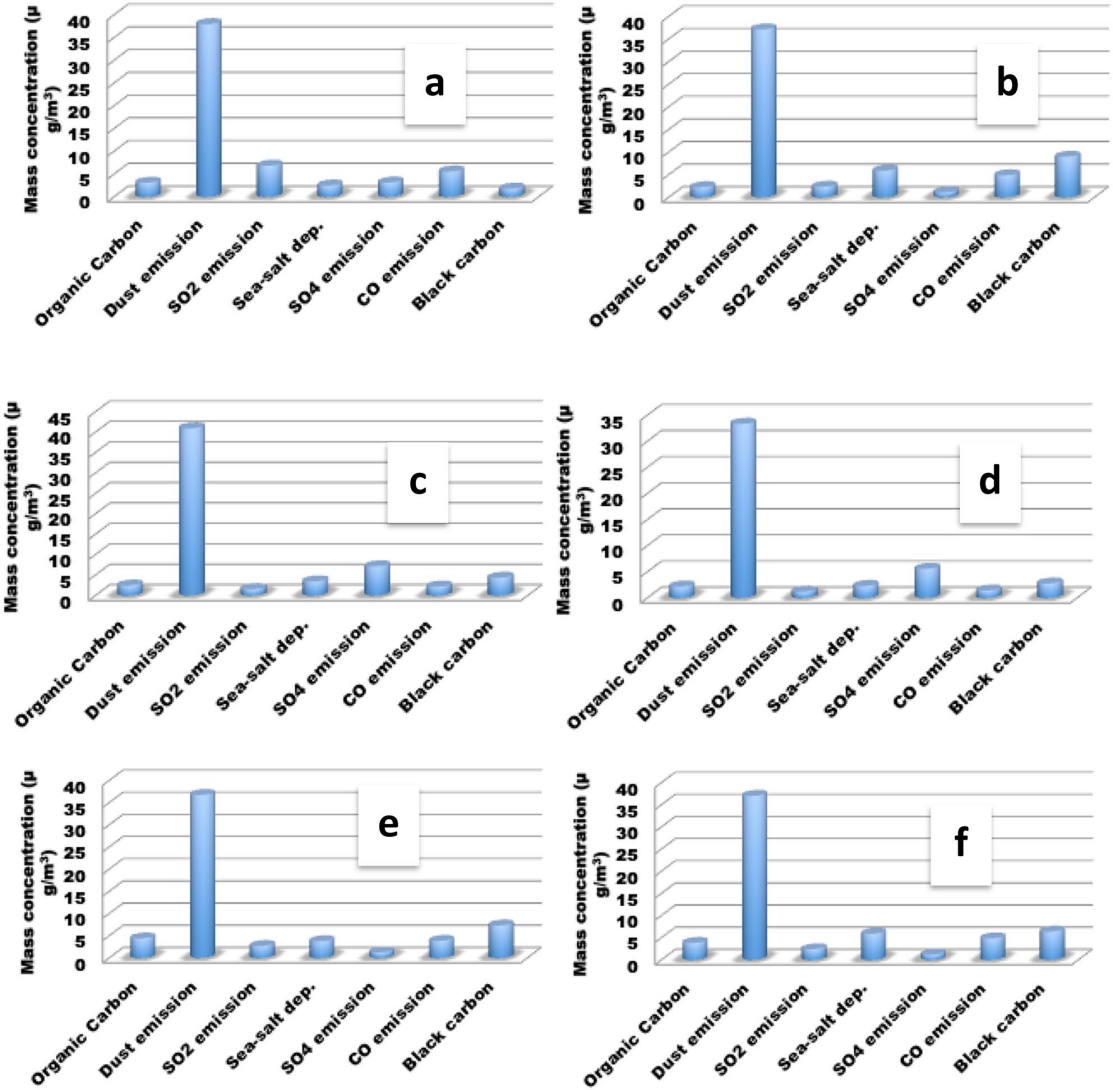
Satellite measurement of major pollutants (**a**) Kano, north-west GZ (**b**) Aba, south-east GZ (**c**) Abuja, north-central GZ (**d**) Borno, north-east GZ (**e**) Lagos, south-west GZ (**f**) Port Harcourt, south-south GZ.

In this study, it was observed that dust pollution had the highest mass concentration among other pollutants (see Fig. 4) according to satellite measurements via MERRA-NASA. Dust pollution in Nigeria is mainly from Sahara and many uncompleted road networks across the nation^45^. Figure 4 shows that dust emission in selected location in Nigeria is generally above 35 g/m^3^. Abuja has the highest dust emission with 39.54 g/m^3^ (Fig. 4c). Ehrmann et al.^48^ reported that 50% of the total desert dust found in the atmosphere is from the Sahara dust influx. Some of the dust content comes from building construction, farm activities, road construction, human activities, industrial emissions (such as cement dust), dust storm, and whirlwind. The Sahara dust is the most prominent among all, as it is believed to be maximum during harmattan. The particulates are borne by the African continental trade wind system, which blows in the northeast direction. In the Nigerian landmass context, the African continental trade wind system blows from north to south; hence, it deposits more dust in the north than in the southern parts. Most time, harmattan dust reaching the southern parts are more PM_2.5_ than PM_10_. Minka et al.^49^ reported that about 400–1200 kg/ha and 100–400 kg/ha dust particles are deposited in the north and south of Nigeria. Pure harmattan dust is mainly composed of quartz; however, due to the source of dust particulates, its chemical constituents are determined from the conditions at which it is generated.

For example, its reaction with atmospheric gases can make it have sulfate, ammonium, nitrates, and organic constituents. Sometimes, it may have heavy metal components, etc. For example, several researchers have proven that dust in both the north and south of Nigeria is made up of heavy metals such as Si, Ti, Al, Fe, V, K, Mg, Ca, Na, Cl, Cu, Pb, Zr, Zn, Br, Mg, As, Se, Cd and Mn^50–53^. Ogundele et al.^54^ had reported that almost the same heavy metals could be found in indoor dust.

Considering the significant pollutions from gaseous particulates such has dust, organic carbon, SO_2_ emission, sea-salt depositions, SO_4_ emissions, and carbon monoxide that is displayed in Fig. 4, some pollutants are significantly high at certain location. SO_2_ and SO_4_ emissions was highest in Kano and Abuja with mass concentration of 5.2 g/m^3^ and 5.1 g/m^3^ respectively. Black carbon emission was found to be high in southern Nigeria with Aba, Lagos and Port Harcourt having mass concentration of 6.8 g/m^3^, 5.3 g/m^3^, and 5 g/m^3^ respectively. Though black carbon was seen to be comparatively low i.e., < 6 g/m^3^, carbon monoxide was found to be significant in big cities as Kano, Lagos, Aba and Port Harcourt with concentrations 4.6 g/m^3^, 2.7 g/m^3^, 3 g/m^3^ and 4 g/m^3^ respectively. These results can be juxtaposed with maternal mortality data presented by Midwives Service Scheme in Nigeria (Fig. 1b) and MNCH2^55^.

**Figure 5:**
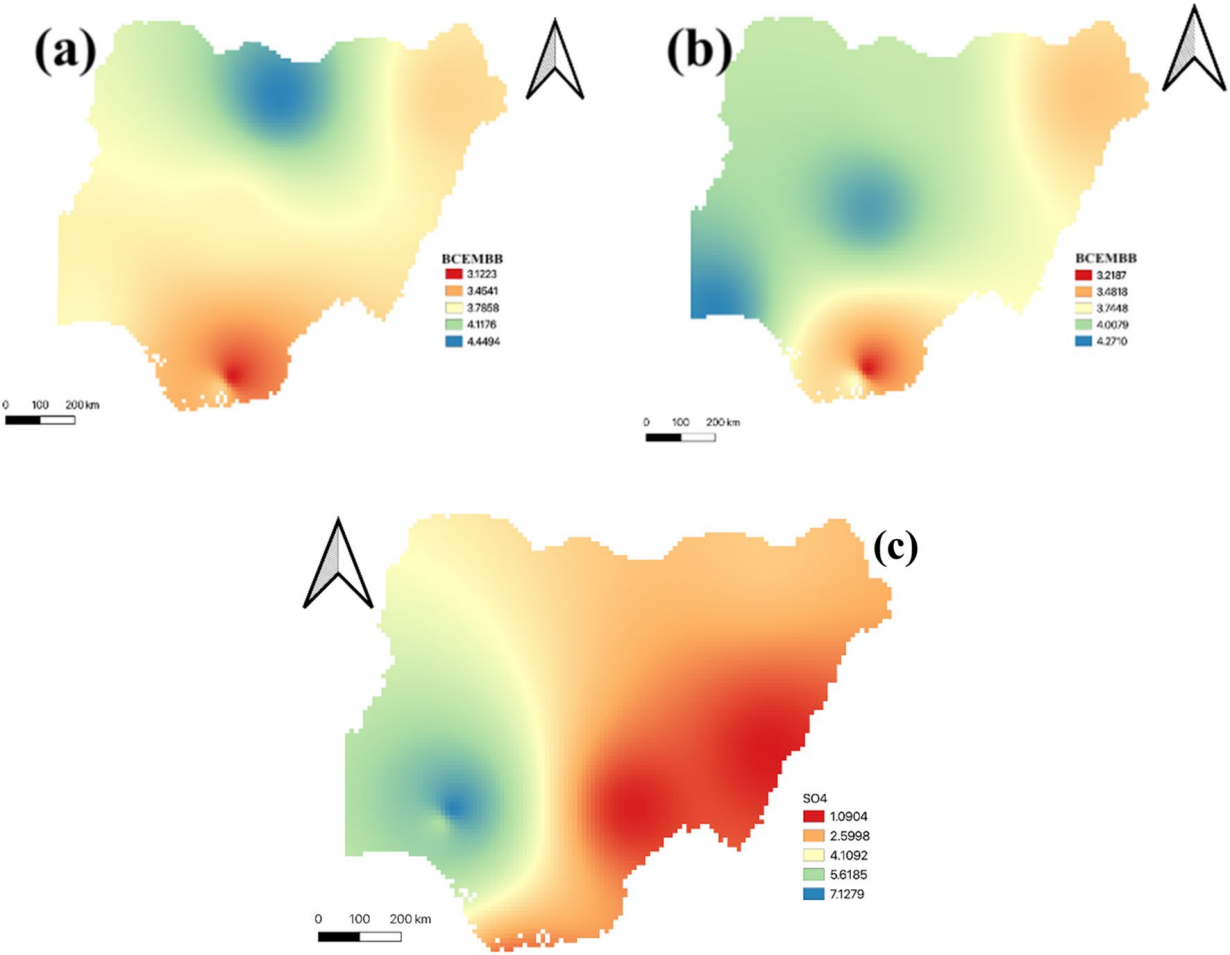
State of black carbon and sulphate particulates (**a**) Black Carbon Biomass Burning Emissions (2010–2014) (**b**) Black Carbon Biomass Burning Emissions (2015–2019) (**c**) Sulphate aerosol.

Aba had significant pollutants such as co emission, black carbon, dust emissions, and sea-salt deposition. Abuja and Borno were seen to have almost trend of pollutants variations within 10 years. Dust, sulphate, and black carbon depositions were more prevalent in both locations. Lagos and port Harcourt had almost same trend as Abuja and Kano except for the relatively high emission of organic carbon. Generally, it can be observed that aside from dust deposition, fossil fuel burning contributes significantly. Figure 5a shows the averages of 5 years (2010–2014) black carbon biomass burning emission dataset with the highest emissions in Kano, Jigawa, and Kastina whose concentration was > 4.2 kgm^−2^ s^−1^. Abdulkadir and Rainis^56^ had been able to validate the significance of this particulates in maternal deaths. Figure 5b shows the averages of the second 5 years dataset (2015–2019) of the black carbon biomass burning emission which was a little reduced by 0.21 kgm^−2^ s^−1^. It was observed that spatial distribution in both north-central and south-west geopolitical zones showed significant emissions that is greater than 4.2 kgm^−2^ s^−1^. The south-south and north-east zones showed stable emission (< 3.1 kgm^−2^ s^−1^) within 10 years.

It was observed that the prevalence of this emission is more significant in the dry season. This trend may be due to farming activities, refuse and bush burning. Akinyemi et al.^57^ showed that the ventilation of locations that is determined by the wind system may be a determining factor in the continuous presence of particulates in the atmosphere. This could be inferred that the wind system in affected locations determines how long pregnant mothers may be at risk. The spatial analysis of the sulphate aerosols across is Nigeria is presented in Fig. 5c below with sulphate aerosols concentration of > 7.1 kgm^−2^ s^−1^ in the south-west and parts of north-central.

It is observed that the concentration of the sulphate particles is diagonally decreasing from south-west to north-east within 10 years. This result was observed to be consistent each year i.e., showing that the cause may likely be localized due to industrial pollution which may be linked to maternal deaths in south-west Nigeria^50,58^.

### Spatial analysis of dust deposition

**Figure 6:**
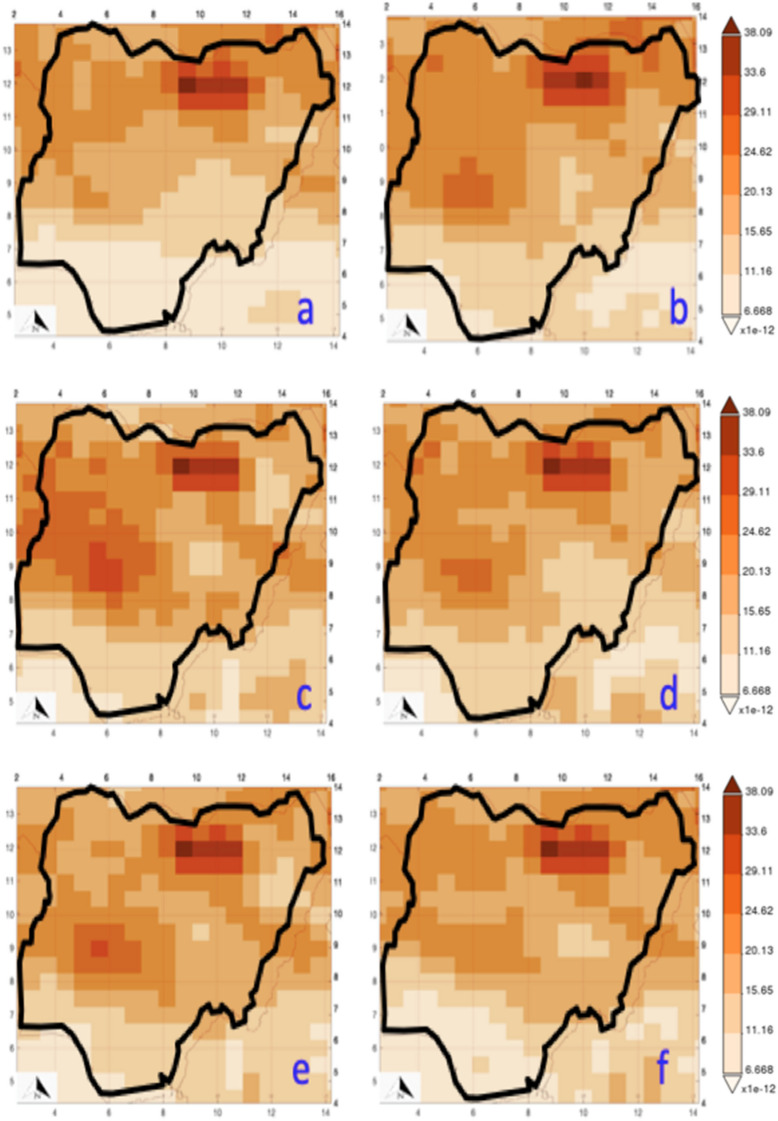
Dust dry deposition (**a**) 2010 (**b**) 2014 (**c**) 2015 (**d**) 2016 (**e**) 2017 (**f**) 2019.

The dust deposition over Nigeria is presented in Fig. 6. Figure 6 was produced using codes and satellite image described in Ref^59^. 6 years dataset (2010, 2014, 2015, 2016, 2017, and 2019) was selected out 10 years available dataset. There were consistent dust depositions at the north-east GZ for the years selected. The highest concentration points (whose dust deposition is above 38 μm^−2^ s^−1^) were observed to shift yearly between Kano (north-west) and Jigawa (north-west). Aside from the Sahara dust influx, the north of Kano had higher industries, construction work, and human activities, as presented in the spatial analyses. With this observation, pregnant women, there are likely to have more respiratory challenges. This scenario explains the main factor for the high maternal mortality rate in Kano to be 1025 deaths per 100,000 live births despite the improved medical services^55^.

**Figure 7:**
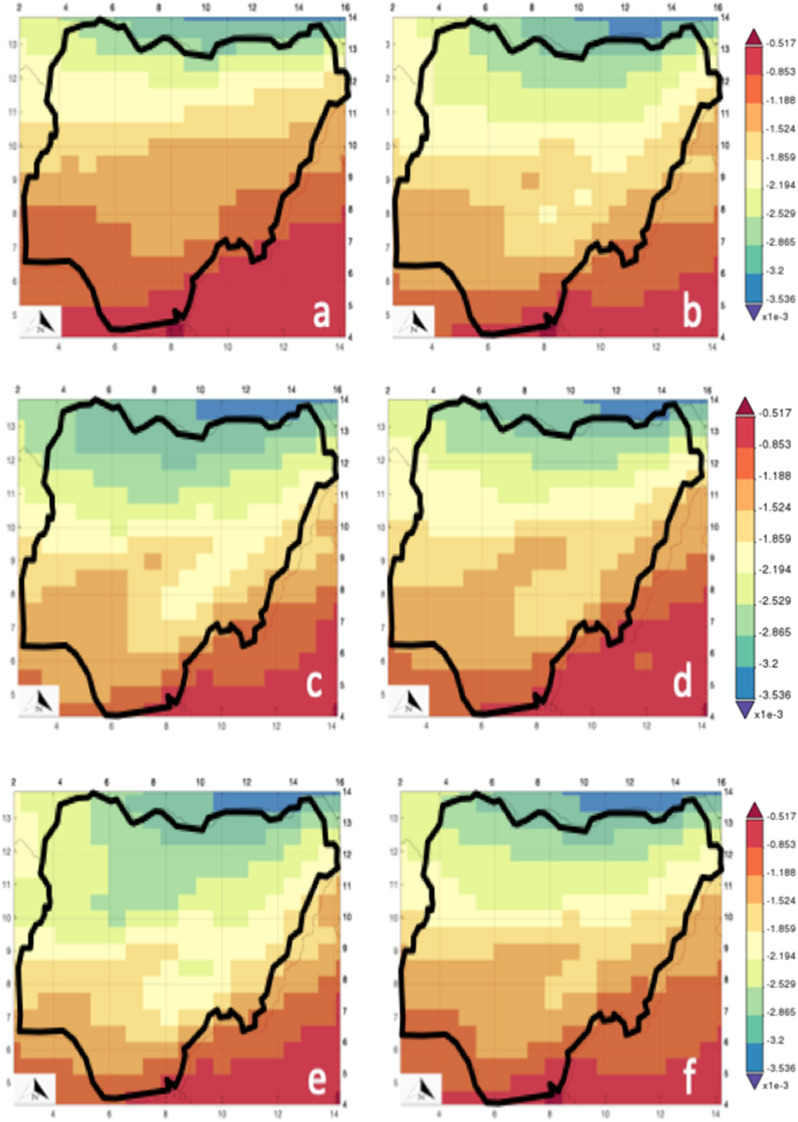
Dust column u-wind mass flux (**a**) 2010, (**b**) 2014, (**c**) 2015, (**d**) 2016, (**e**) 2017 (**f**) 2019.

Secondly, it is observed that dust depositions are noticeable over the three northern geopolitical zones with dust depositions greater than 34 kgm^−2^ s^−1^, suggesting that the deposition is mainly controlled by the dust column u-wind mass flux (> 0.0003.2 kgm^−1^ s^−1^as revealed in Fig. 7) and the particulate matter's sizes (within PM_2.5_ and PM_10_). Like Kano, another prominent dust deposition location is Niger state (north-central GZ). Aside from the Sahara dust influx, the location is characterized by an untarred road network that is believed to be > 65%. The untarred road network is plied regularly as the location is the connecting route for traders and working populations from neighbouring locations such as Abuja.

The southern parts of Nigeria were observed to have less concentration of dust deposits (< 11 μm^−2^ s^−1^), which further confirms that the Sahara dust influx is the primary type of pollution across the country^49^. Juxtaposing dust emission (Fig. 4) and dust deposition (Fig. 6), it can be seen that the influence of the dust column v-wind mass flux which is > 0.00035 kgm^−1^ s^−1^ plays significant role in both parameters. Also, it is observed that dust deposition in southern Nigeria was high between 2014 (11.2 kgm^−2^ s^−1^) and 2016 (11.32 kgm^−2^ s^−1^); and relatively low in 2010 (8.28 kgm^−2^ s^−1^) and 2019 (8.43 kgm^−2^ s^−1^). The south's low dust deposition could be attributed to the vegetation that serves as a windbreaker for the northeast wind conveying dust from Sahara. However, with dust deposition of > 10 μm^−2^ s^−1^, pregnant women and infants are more at risk due to the heavy metal content and the radioactive particulates in the inhaled dust.

It was closely observed that a section in the north-central GZ was consistently low (6.79 kgm^−2^ s^−1^)despite the prevailing Sahara dust influx. The location is identified as Plateau state. This occurrence may be ascribed to its topography i.e.; altitude ranges from around 1200 m (3900 ft) to a peak of 1829 m (6001 ft) above sea level. Plateau's maternal mortality rate is given as 905 per 100,000 live births^60^. Likewise, in the south, Bayelsa state (south-south GZ) consistently had low dust deposits (6.53 kgm^−2^ s^−1^). Its average elevation is 18 m above mean sea level. In other words, it has been discovered that dust deposition depends on topography; hence, pregnant women within extreme elevations may be safer.

The spatial analysis of the dust column u-wind mass flux (DCUMF) is presented in Fig. 7. Figure 7 was produced using codes and satellite image described in Ref^59^. The diagram shows that DCUMF is higher in the north than in the south of Nigeria. DCUMF trend shows that it is decreasing from north (> 0.00034 kgm^−1^ s^−1^) to south (> 0.000052 kgm^−1^ s^−1^). This result shows that the transport of dust from Sahara by the northeast trade wind is strongly supported by the zonal wind speed that varies yearly. The comparison between the dust deposition and DCUMF clearly explains why there is a significant yearly variation that depends on the atmosphere's volume of dust. In other words, as lighter dust is carried from north to south, it combines with the localized dust in the south and deposits at varying locations, as seen in Fig. 7. More significantly is the possibility that the lighter Sahara dust might have chemically reacted with the localized dust to yield more dangerous products such as heavy metallic dust and/or radioactive dust particulates. Hence, the danger might not be inhalation, but the extensive reaction that occurs with the body since pregnant women are the most vulnerable because of their reduced lung capacity. WHO^61^ reported that in 2012, lower respiratory infections were the leading cause of death that was estimated to 290.2 thousand.

**Figure 8:**
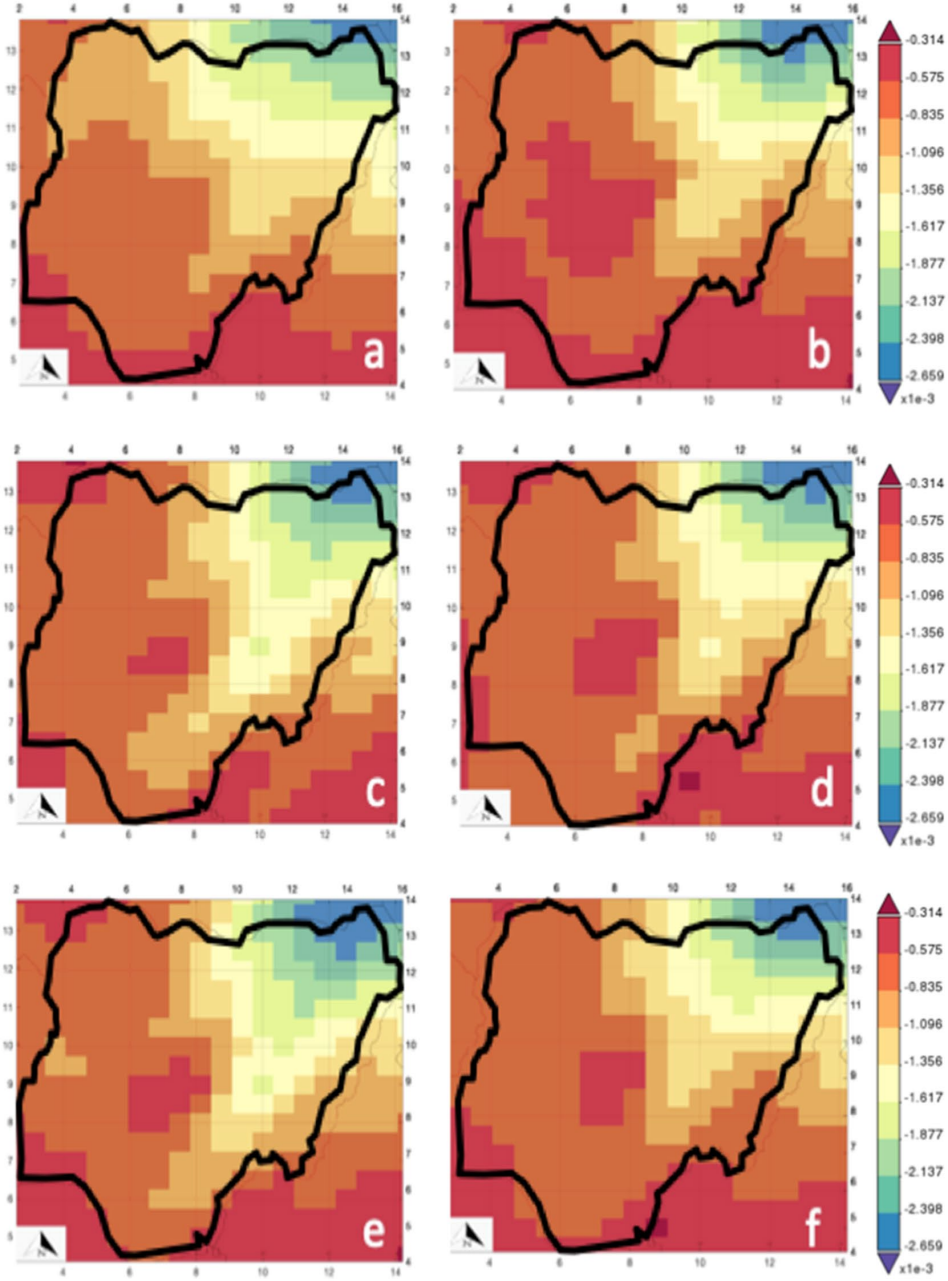
Dust column v-wind mass flux (**a**) 2010, (**b**) 2014, (**c**) 2015, (**d**) 2016, (**e**) 2017 (**f**) 2019.

The spatial analysis of the dust column V-wind mass flux (DCVMF) is presented in Fig. 8. Figure 8 was produced using codes and satellite image described in Ref^59^. DCVMF increases from east (> 0.000265 kgm^−1^ s^−1^) to west (> 0.0000314 kgm^−1^ s^−1^) with a higher magnitude at every other year. The meridional velocity seems to have a turning effect around the north-central geopolitical zones and the unnecessary parts of south-west and south-south geopolitical zones. This occurrence yields a lower dust column in the affected region. When the dataset is combined with dust deposition, more localized dust is emitted in the north-central. Most time, localized dust emission may increase as the indoor dust. This assertion is corroborated by the work of Ogundele et al.^54^ at the extreme parts of the south-west GZ. Both the DCVMF and DCUMF affirm that Borno and its surrounding environs may have the highest dust column mass influx. This result explains the high maternal mortality rate in the region.

### Statistical analysis of dust deposition in Nigeria

**Figure 9:**
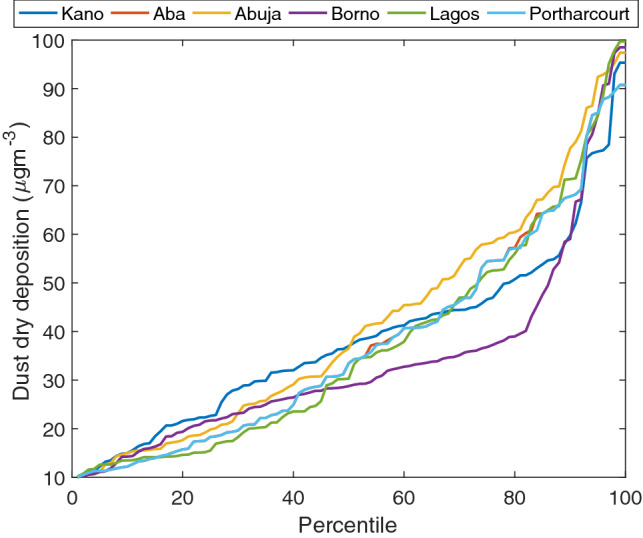
Percentile classification of dry dust deposition.

The percentile plot of the dry dust deposition (DDD) for the selected locations in each geopolitical zone is presented in Fig. 9. The percentile trend is said to be parabolic. This trend means that all the regions have a common primary source of pollution, i.e., Sahara dust. The range of datasets falls at a particular frequency that shows its coherence. For example, in descending order at the 25th percentile, the DDD are Kano (22.67 μgm^−3^), Borno (21.71 μgm^−3^), Abuja (19.89 μgm^−3^), Aba (18.34 μgm^−3^), Port Harcourt (18.34 μgm^−3^), and Lagos (15.85 μgm^−3^). At the 25th percentile, the dataset's background nature can be known as it corroborates the spatial analysis in Fig. 4. Taking the DDD at 75th percentage, it was observed that its value is given as Abuja (58 μgm^−3^), Aba (54.12 μgm^−3^), Port Harcourt (54.12 μgm^−3^), Lagos (51.91 μgm^−3^), Kano (46.41 μgm^−3^), and Borno (36.72 μgm^−3^). The higher percentile depicts the localized DDD trend, i.e., dust generated from various activities aside from the Sahara dust influx. The difference between the 75th and 25th percentile gives a clear picture of the dataset's coherence for 10 years. Lower values show better coherence of the dataset as presented in Borno (15 μgm^−3^), Kano (23.74 μgm^−3^), Aba (35.79 μgm^−3^), Port Harcourt (35.79 μgm^−3^), Lagos (36.06 μgm^−3^), and Abuja (38.12 μgm^−3^). This result corroborates the spatial analysis for dust column v-wind mass flux that the meridional velocity over north-central and extreme parts of south-south and south-west favour lower dust deposition.

The statistical parameters of mean, standard error, variance, and standard deviation for each location are displayed in Table 3. Unlike the percentile classification, the standard error, variance, and standard deviation show that the dataset's coherence is in descending order of Kano, Borno, Aba, Port Harcourt, Lagos, and Abuja. The highest mean dust deposition was found in Abuja (40.97) which does not in any way mean that other locations do not have higher daily dust deposition within the 10 years considered. It was observed that the though Aba had higher DDD mean (37.14) than Lagos, the standard error in Lagos is higher because of DDD spikes at certain times of the year. Over 10 years, the DDD mean shows that in descending order, Abuja, Kano, Aba, Port Harcourt, Lagos, and Borno. This result shows a rough estimation of local and Sahara dust coagulation in the area. Hence, despite the general influence of Sahara dust, the localized dust emission's impact is significant.

**Table 3:** Statistical parameters of selected locations.

	Kano	Aba	Abuja	Borno	Lagos	P/Harcourt
Mean	37.96123	37.13633	40.96812	33.35485	36.74592	37.03275
Std. error	1.649851	2.019057	2.151816	1.780298	2.117748	2.011084
Variance	326.6411	489.1908	555.6373	380.3352	538.1828	485.3351
Stand. dev	18.07322	22.11766	23.57196	19.50218	23.19877	22.03032

The Pearson correlation of the selected locations in the geopolitical zones is presented in Table 4. The significant correlation was observed only for Port Harcourt and Aba. Dust deposition shows a significant correlation in south-west and south-south geopolitical regions of Nigeria. It was also observed that the dataset in other regions (south-west, north-central, north-west, north-east) did not have significant correlations. This result simply means that there are significant local dust depositions than Sahara dust deposition. Hence, there will be higher indoor dust in the north-central GZ in Nigeria. This result explains the relatively high maternal mortality rate proposed by Ref^60,61^.

**Table 4:** Pearson correlation of selected locations.

	Kano	Aba	Abuja		Borno	Lagos	P/Harcourt
Kano		4.90E−01		0.21148	2.91E−07	0.023483	5.34E−01
Aba	**−0.063567**			0.48108	7.88E−01	9.53E-04	3.05E−01
Abuja	**−0.11489**	**−0.064929**			0.37073	0.00079543	0.46816
Borno	0.44785	**−0.024825**		**−0.082435**		0.11323	8.12E−01
Lagos	0.20673	0.29785		**−0.30218**	0.14534		8.95E−04
P/Harcourt	**−0.057321**	0.9993		**−0.066853**	**−0.021902**	0.29937	

Significant values are in bold.

### Risk analysis of inhaled dust

**Figure 10:**
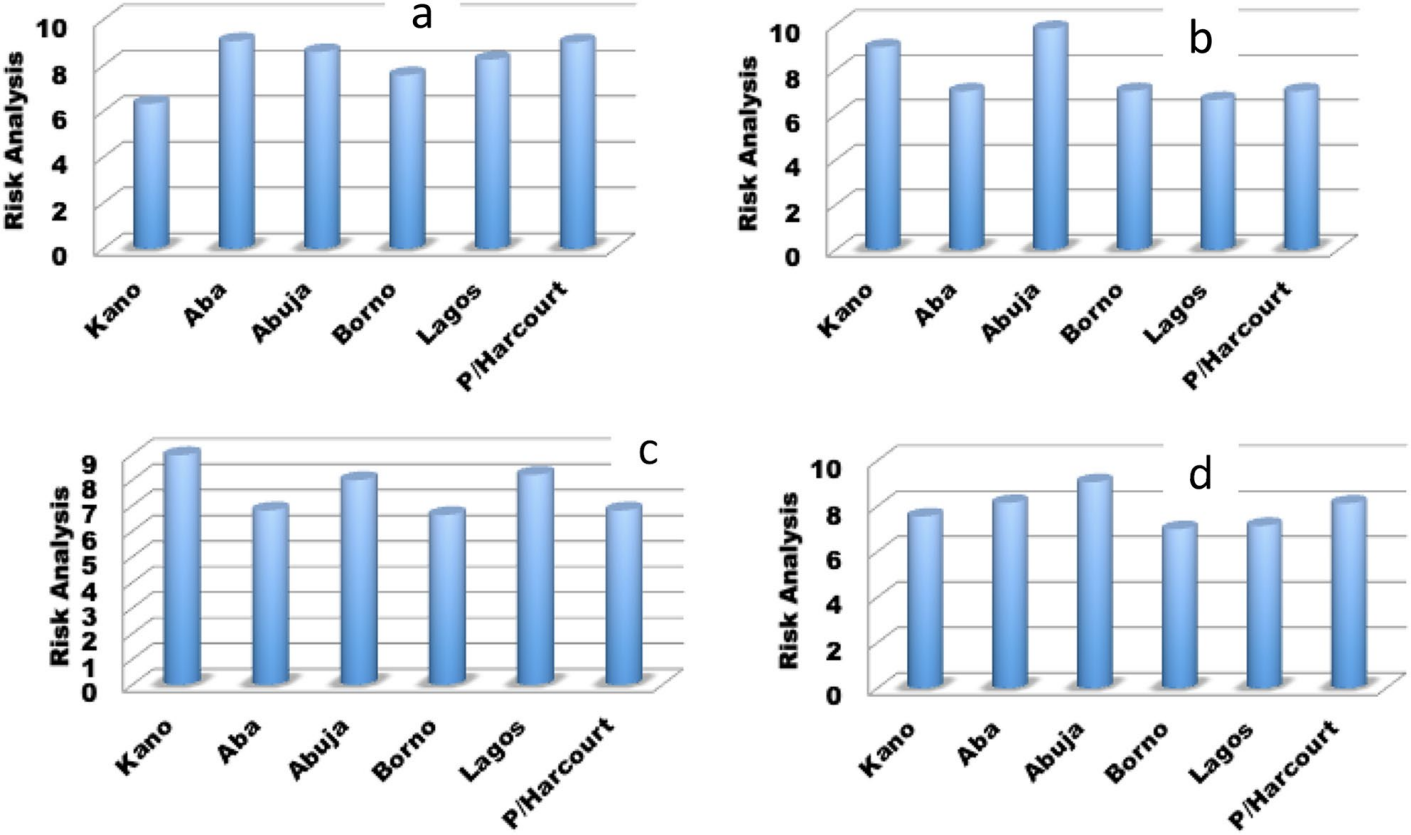
Risk analysis (**a**) early dry season-SON (**b**) late dry season-DJF (**c**) early wet season-MAM (**d**) late dry season-JJA.

Using the USEPA risk analysis calculation, the risk for the four seasons i.e. early dry season (September, October, and November), late dry season (December, January, and February), early wet season (March, April, and May), and late wet season (June, July, and August) as presented in Fig. 10. The average mass concentration for each season and location was used to estimate the risk assessment. It was observed that the risk accrue to dust inhalation in the early dry season is more pronounced in Aba (south-east) and Port Harcourt (south-south) whose average daily uptake is 9.3 μg/kg/day (Fig. 10). Abuja (north-central), Lagos (south-west), Borno (north-east), and Kano (north-west) had average daily uptake that is greater than 5.8 μg/kg/day. In the late dry season, Abuja had the highest average daily uptake of 9.6 μg/kg/day. Kano, Borno, Aba, Port Harcourt, and Lagos had average daily uptake that is greater than 6 μg/kg/day. In the early wet season, Kano had the highest average daily uptake of 8.45 μg/kg/day. Lagos, Abuja, Aba, Port Harcourt, and Borno had average daily uptake that is greater than 6 μg/kg/day. In the late wet season, Abuja had the highest average daily uptake of 8.3 μg/kg/day. Aba, Port Harcourt, Kano, Lagos, and Borno had average daily uptake that is greater than 6.28 μg/kg/day. These results further corroborate the assertion in Fig. 1b. Several pieces of literature have shown varying exposure limits for different scenarios. For example, the occupational exposure limits (OEL) for respirable dust and respirable crystalline silica (RCS) for mining workers are given as 1.5 mg/m^3^ and 0.05 mg/m^3^, respectively^62^. HSE^63^ recommended the exposure limits to coal dust as 1 mg/m^3^ over a working lifetime of 40 years. TUC’s recommended that precautionary dust standard as 2.5 mg/m^3^ and 1 mg/m^3^ (TUC, 2011). Considering different limits mentioned above, the risk assessment is high for all regions ^64–67^.

**Figure 11:**
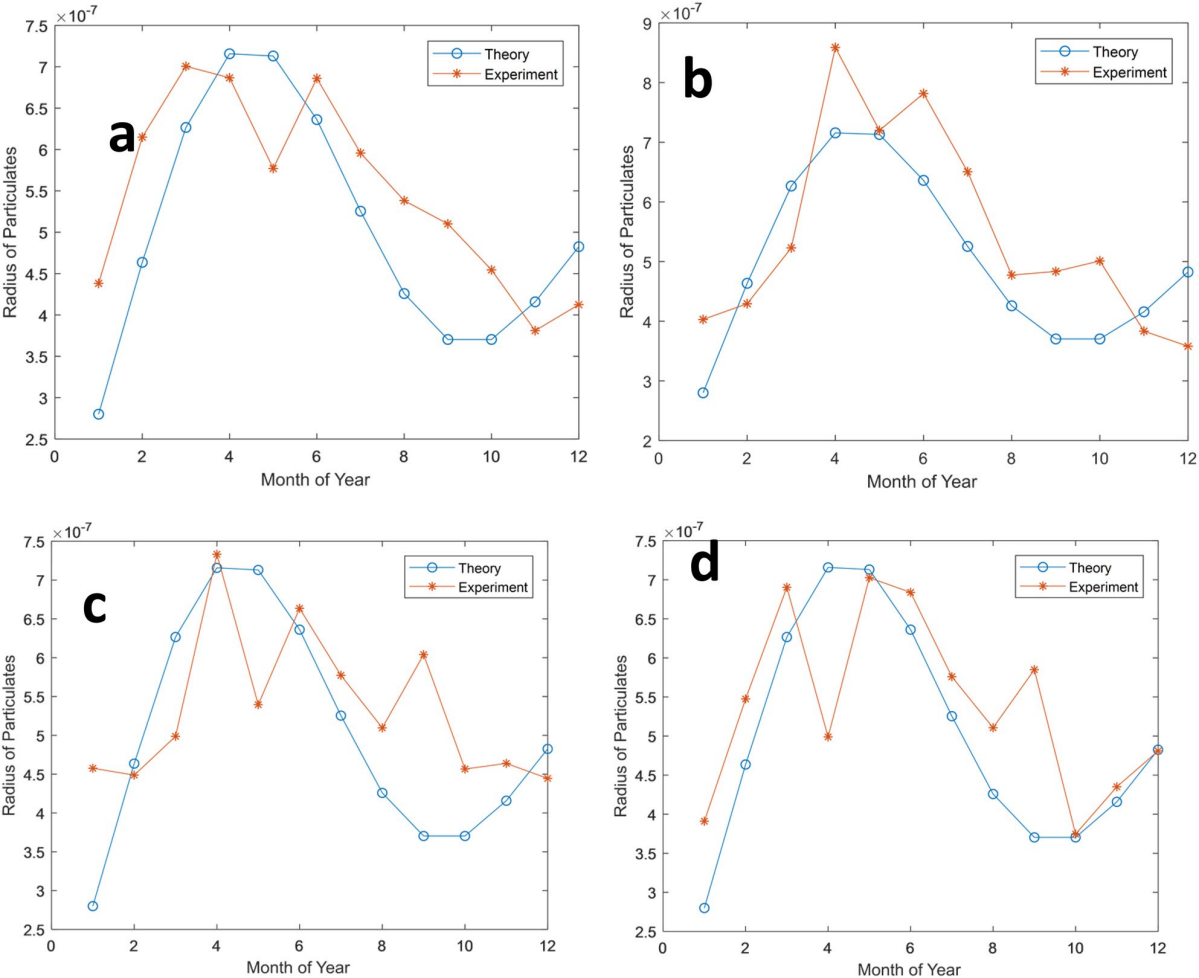
Dust deposition model in human lungs.

The second part of the risk analysis is the theoretical estimation of the deposition of dust particulates in pregnant women's lungs. The radius of the particulates was estimated and modelled using Eq. (8), as presented in Fig. 11. It was observed that there is a direct proportionality between the size of outdoor or indoor dust content and its deposition in the human lungs. It was found out that the dust deposition in the lungs is higher in the early wet season and lowest in the early dry season. This assertion is realistic, as the early rains would have pull-down heavier or larger dust particles, i.e., leaving the lighter particles for inhalation. The seasonal dataset of dust depositions also confirms this hypothesis. However, the risk analysis shows that these effects vary from one location to another. Also, each location's risk analysis is unpredictable, as its localized dust varies according to the human population, activities, and migrations. In other words, pregnant women can reduce dust deposition into their lungs by using facemask outdoor and reducing dust emission indoors during the highlighted seasons.

The third segment estimates the radiological risk for radionuclide dust content, as listed by previous authors within the research area. Six elements were considered, i.e., Cl, Mn, Pb, Se, Zn, and Zr. The ERICA dose rate of 10 μGyh-1 was adopted in an air medium of the IAEA SRS-19 model. The concentration ratio for each element is given as 0.008 Bq/m^3^ (Cl), 0.00234 0.008 Bq/m^3^ (Mn), 0.0374 0.008 Bq/m^3^ (Pb), 0.18 0.008 Bq/m^3^ (Se), 0.299 0.008 Bq/m^3^ (Zn), and 0.000254 0.008 Bq/m^3^ (Zr). The values were sourced from literature for dust particulates containing each radionuclide. The result of the radiological risk for pregnant women is presented in Tables 3 and 4.

Table 5 presents the values of heavy metals and Table 6 presents the values of radionuclides in atmospheric dust in selected parts of Nigeria. Tables 6 and 7 were the main parameters used for the simulation of the radiological risk using ERICA. Table 7 presents the dose rate type for external and internal conditions. It is observed that the dose rate is higher when particulates are inhaled into the lungs Cl, Mn, Pb, Se, and Zr. Contrary to the above, the dose rate is lower when Zn radionuclide is inhaled into the lungs. Like the mass concentration, the total dose rate is on the micro-scale. Hence, the continued inhalation of this radionuclide for a long time will certainly constitute a health hazard. The dose conversion coefficient of the radionuclide is presented in Table 8. Taking a cue from the International Commission's inhalation standards on Radiological Protection (ICRP) for the under listed radionuclide^68^. It can be inferred that it is slightly high if it is inhaled into the lungs. In other words, looking at the deposition pattern into the human lungs, it can be inferred the lung capacity of the pregnant woman under this condition has been compromised before pregnancy; hence, at pregnancy, it is easier to show various signs of respiratory malfunctioning. In other words, though there is a lot to be done aside from providing health facilities. Environmental authorities owe the public the duty to create public awareness of sources and the impact of dust deposition to reduce clinical cases. Another observation is that the dose conversion coefficient tends to increase within internal organs than external habitants.

**Table 5:** Heavy metal contents in atmospheric dust in selected parts of the study area.

No	Heavy metal	Concentration (μg/g)	Location	References
1	Pb	32.33	Akure (Southwest	^69^
		2.90–8.73	Kano (Northwest)	^70^
		2.34–10.17	Ilorin (Northcentral)	^71^
		22.29–95.485	Kaduna (Northwest)	^72^
		0.121–0.832	Jos (Northcentral)	^73^
		2.34–20.12	Yobe (Northeast)	^74^
2	Zn	72.53	Akure (Southwest	^69^
		160.5–270.13	Kano (Northwest)	^70^
		0.696–1.712	Jos (Northcentral)	^73^
		2.34–32.44	Yobe (Northeast)	^74^
3	Fe	75.46–224.76	Kano (Northwest)	^70^
		28.6–45.40	Ilorin (Northcentral)	^71^
		0.171–1.081	Jos (Northcentral)	^73^
		3.45–34.23	Yobe (Northeast)	^74^
4	Mn	3.88–6.80	Kano (Northwest)	^70^
	Mn	0.002–0.056	Jos (Northcentral)	^73^
	Mn	1.09–6.34	Yobe (Northeast)	^74^
5	Mg	5.00–9.82	Kano (Northwest)	^70^
6	As	0.19–3.98	Yobe (Northeast)	^74^
7	Ca	104.33–202.90	Kano (Northwest)	^70^
8	Co	0.88–7.87	Yobe (Northeast)	^74^
9	Cu	2.75–189.50	Kano (Northwest)	^70^
		0.19–1.99	Ilorin (Northcentral)	^71^
		0.025–0.571	Jos (Northcentral)	^73^
		0.225–1.280	Kaduna (Northwest)	^72^
		1.98–10.34	Yobe (Northeast)	^74^
10	Cr	1.75–62.53	Kano (Northwest)	^70^
		0.019–0.111	Jos (Northcentral)	^73^
		16.670–31.895	Kaduna (Northwest)	^72^
		1.23–8.22	Yobe (Northeast)	^74^
11	Cd	0.001–0.38	Ilorin (Northcentral)	^71^
	Cd	0.002–0.438	Jos (Northcentral)	^73^
	Cd	0.280–1.900	Kaduna (Northwest)	^72^
	Cd	1.54–9.23	Yobe (Northeast)	^74^
12	Ni	0.476–1.619	Ilorin (Northcentral)	^71^
	Ni	0.021–0. 478	Jos (Northcentral)	^73^
	Ni	1.560–5.880	Kaduna (Northwest)	^72^
	Ni	4.34–14.34	Yobe (Northeast)	^74^

**Table 6:** Radionuclide contents in atmospheric dust in selected parts of the study area.

No	Radionuclide	Concentration	Location	Reference
1	^226^Ra	12.61–47.81	Ogun (Southwest	^73^
		52.16	Plateau (Northcentral)	^75^
2	^232^Th	34.67–66.91	Ogun (Southwest	^73^
		29.76	Plateau (Northcentral)	^75^
3	^40^K	320.82–680.50	Ogun (Southwest	^73^
		510.82	Plateau (Northcentral)	^75^

**Table 7:** Inhaled dose rate of radionuclide deposition in the human lungs.

Elements	External Dose Rate [µGy h^-1^]	Internal Dose Rate [µGy h^-1^]	Total Dose Rate [µGy h^-1^]	Activity Concentration in air [Bq kg^-1^ d.w. or Bq m^-3^]
	Isotope	Isotope	Isotope	
Cl-36	1.2E−10	0.00000272	2.72012E−06	0.008
Mn-54	1.9656E−07	5.0286E-06	5.22516E−06	0.00234
Pb-210	2.8798E−09	0.00000442	4.42288E−06	0.0374
Se-79	0	5.5488E-07	5.5488E−07	0.18
Zn-65	1.77708E−05	3.40835E-06	2.11791E−05	0.299
Zr-95	1.8796E−08	0.00000544	5.4588E−06	0.000254

**Table 8:** Dose conversion coefficient of radionuclide in the human lungs.

Elements	Concentration ratio (CR) [Bq m^−3^ air ]	Dose conversion coefficient of int-low-beta [µGy h^−1^ pr. Bq kg^−1^]	Dose conversion coefficient of int-beta-gamma [µGy h^−1^ pr. Bq kg^−1^]	Dose conversion coefficient of ext-on-soil-beta-gamma [µGy h^−1^ pr. Bq kg^−1^]
	Nuclide	Isotope	Isotope	Isotope
Cl	7	0	0.00016	0.000000015
Mn	0.00249	0.0000029	0.0002871	0.000084
Pb	0.037355054	0.000005	0.000245	0.000000077
Se	0.18611226	0.00000032	0.00003168	0
Zn	2.99000783	2.69992E−06	0.000192391	5.9434E−05
Zr	0.000254	0	0.00032	0.000074

The risk quotient is given as 0.00000396, while the total dose rate per organism is given as 0.0000396 µGy h^−1^. The implication of these results is the following: minor decrease of body weight, a minor decrease of peripheral blood cells, a minor decrease of peripheral white blood cells, and a moderate decrease of life span.

## Conclusion

This study has proven that air pollution is salient in maternal mortality in Nigeria. The results obtained in this study corroborates the dataset given by the Midwives Service Scheme in Nigeria. Also, it was shown via several techniques that aside from the Sahara dust influx, the emission of localized dust in each location in the country is very significant due to the human population, activities, and migration. The release of the localized dust content sometimes reacts with the Sahara dust particles to form a dust column that has heavy metal and/or radionuclide particles; hence, when the dust is inhaled, the risk is not limited to the volume of dust inhaled but to the extensive reaction in the body to cause respiratory disorder or other known diseases in pregnant women. Dust deposition over each geopolitical zone was found to be proportional to the maternal mortality rate. This discovery makes controlling whatever type of dust a national priority, as it was discovered that the level of cumulative dust content in some parts of the country is more than geographical locations close to the Sahara Desert.

It was clearly shown that both natural and artificial anthropogenic pollution constitute air hazards across the country. Also, it was shown that the risk factor varies according to location. It is recommended that pregnant women could reduce dust deposition into their lungs by using facemask when outdoor and using pollutants alarm sensors within indoor environment. In other words, though there is a lot to be done aside from providing health facilities. Environmental authorities owe the public the duty to create public awareness of sources and the impact of air pollution to reduce clinical cases.

## References

[1] Pope C, Aruni B, James P, Luid M, Wesley TA, Daniel JC, Timothy EO (2016). Exposure to fine particulate air pollution is associated with endothelial injury and systemic inflammation. Circ. Res..

[2] Checa Vizcaíno MA, González-Comadran M, Jacquemin B (2016). Outdoor air pollution and human infertility: A systematic review. Fertil Steril..

[3] Patel AB, Meleth S, Pasha O, Goudar SS, Esamai F, Garces AL, Chomba E, McClure EM, Wright LL, Koso-Thomas M (2015). Impact of exposure to cooking fuels on stillbirths, perinatal, very early and late neonatal mortality—A multicenter prospective cohort study in rural communities in India, Pakistan, Kenya, Zambia and Guatemala. Matern. Health Neonatol. Perinatol..

[4] Brunekreef B, Holgate ST (2002). Air pollution and health. Lancet.

[5] Stanek LW, Brown JS, Stanek J, Gift J, Costa DL (2011). Air pollution toxicology—A brief review of the role of the science in shaping the current understanding of air pollution health risks. Toxicol. Sci..

[6] Thornburg J, Islam S, Billah SM, Chan B, McCombs M, Abbott M, Alam A, Raynes-Greenow C (2022). Pregnant women’s exposure to household air pollution in rural Bangladesh: A feasibility study for poriborton: The CHANge Trial. Int. J. Environ. Res. Public Health.

[7] Maghbooli Z, Hossein-nezhad A, AdabiE A-P, Sadeghi M, Mohammad-nabi S (2018). Air pollution during pregnancy and placental adaptation in the levels of global DNA methylation. PLoSONE.

[8] Perera FP, Jedrychowski W, Rauh V, Whyatt RM (1999). Molecular epidemiologic research on the effect of environmental pollutants on the fetus. Environ. Health Perspect..

[9] Agay-Shay K, Friger M, Linn S, Peled A, Amitai Y, Peretz C (2013). Air pollution and congenital heart defects. Environ. Res..

[10] Arroyo V, Díaz J, Carmona R, Ortiz C, Linares C (2016). Impact of air pollution and temperature on adverse birth outcomes: Madrid, 2001–2009. Environ. Pollut..

[11] Beck S, Wojdyla D, Say L, Betran AP, Merialdi M, Requejo JH, Rubens C, Menon R, Van Look PF (2010). The worldwide incidence of preterm birth: a systematic review of maternal mortality and morbidity. B World Health Organ..

[12] RSilva R, Lichtenfels AJF, Pereir LAA, Saldiva PH (2008). Effects of ambient levels of air pollution generated by traffic on birth and placental weights in mice. Fertil. Steril..

[13] Ahmed SM, Mishra GD, Moss KM, Yang IA, Lycett K, Knibbs LD (2022). Maternal and childhood ambient air pollution exposure and mental health symptoms and psychomotor development in children: An australian population-based longitudinal study. Environ. Int..

[14] Ekeus C, Lindstrom K, Lindblad F (2010). Preterm birth, social disadvantage, and cognitive competence in Swedish 18- to19-year-old men. Pediatrics.

[15] Savitz DA, Elston B, Bobb JF, Clougherty JE, Dominici F, Ito K (2015). Ambient fine particulate matter, nitrogen dioxide, and hypertensive disorders of pregnancy in New York City. Epidemiology.

[16] WHO- World Health Organization Air Quality Guidelines. Global Update. Report on a Working Group Meeting, Bonn, Germany (2005).

[17] Malmqvist E, Rignell-Hydbom A, Tinnerberg H, Björk J, Stroh E, JakobssonK RR, Rylander L (2011). Maternal exposure to air pollution and birth outcomes. Environ. Health Perspect..

[18] Kourtis AP, Read JS, Jamieson DJ (2014). Pregnancy and infection. New England J. Med..

[19] Cervantes-Gonzalez M, Launay O (2010). Pandemic influenza A (H1N1) in pregnant women: impact of early diagnosis and antiviral treatment. Expert Rev. Anti. Infect. Ther..

[20] Habak PJ, Griggs, Jr RP Urinary Tract Infection In Pregnancy. In: StatPearls [Internet]. Treasure Island (FL): StatPearls Publishing (2022) https://www.ncbi.nlm.nih.gov/books/NBK537047/. 30725732

[21] Waikhom SD, Afeke I, Kwawu GS (2020). Prevalence of vulvovaginal candidiasis among pregnant women in the Ho municipality, Ghana: Species identification and antifungal susceptibility of Candida isolates. BMC Pregnancy Childbirth.

[22] Dauby N, Goetghebuer T, Kollmann TR, Levy J, Marchant A (2012). Uninfected but not unaffected: chronic maternal infections during pregnancy, fetal immunity, and susceptibility to postnatal infections. Lancet. Infect. Dis..

[23] WHO Maternal health in Nigeria: generating information for action (2019) https://www.who.int/reproductivehealth/maternal-health-nigeria/en/#:~:text=Nigeria%20is%20also%20the%20country,all%20global%20maternal%20deaths%20happen.&text=In%202015%2C%20Nigeria's%20estimated%20maternal,maternal%20deaths%20during%20that%20year.

[24] Meh C, Thind A, Ryan B (2019). Levels and determinants of maternal mortality in northern and southern Nigeria. BMC Pregnancy Childbirth.

[25] Sageer R, Kongnyuy E, Adebimpe WO (2019). Causes and contributory factors of maternal mortality: evidence from maternal and perinatal death surveillance and response in Ogun state Southwest Nigeria. BMC Pregnancy Childbirth.

[26] World Bank Nigeria - Maternal Mortality Ratio (modeled Estimate, Per 100,000 Live Births) (2017) http://datatopics.worldbank.org/world-development-indicators/.

[27] Okonofua F, Ntoimo L, Ogungbangbe J, Anjorin S, Imongan W, Yaya S (2018). Predictors of women’s utilization of primary health care for skilled pregnancy care in rural Nigeria. BMC Pregnancy Childbirth.

[28] Abimbola S, Okoli U, Olubajo O, Abdullahi MJ, Pate MA (2012). The midwives service scheme in Nigeria. PLoS Med..

[29] QGIS Development Team QGIS Geographic Information System. Open Source Geospatial Foundation Project (2022) http://qgis.osgeo.org".

[30] Countrymeters Nigeria Population. https://countrymeters.info/en/Nigeria (Accessed 22 July, 2022) (2022).

[31] Chukwu TM, Morse S, Murphy R (2022). Poor air quality in urban settings: A comparison of perceptual indicators, causes and management in two cities. Sustainability.

[32] Yakubu OH (2018). Particle (Soot) pollution in port Harcourt Rivers State, Nigeria—Double air pollution burden? Understanding and tackling potential environmental public health impacts. Environments.

[33] Giwa SO, Nwaokocha CN, Kuye SI, Adama KO (2019). Gas flaring attendant impacts of criteria and particulate pollutants: A case of Niger Delta region of Nigeria. J. King Saud Univ. Eng. Sci..

[34] Okedere OB, Elehinafe FB, Oyelami S, Ayeni AO (2021). Drivers of anthropogenic air emissions in Nigeria—A review. Heliyon.

[35] EPA Sources of indoor air pollution—carbon monoxide. Retrieved from: www.epa.gov/iaq/co.html# Accesed July 19 2021 (2006).

[36] Boyles AL, Beverly BE, Fenton SE, Jackson CL, Anne MZ, Jukic VL, Sutherland DD, Baird GW, Collman DD, Ferguson KK, Hall JE, Martin EM, Schug TT, White AJ, Chandler KJ (2021). Environmental factors involved in maternal morbidity and mortality. J. Womens Health.

[37] USEPA Human health risk assessment protocol for hazardous waste combustion facilities, 1988 (1997) http://www.epa.gov/epaoswer/hazwaste/combust/risk.htm

[38] Wilhelm M, Ritz B (2005). Local variations in CO and particulate air pollution and adverse birth outcomes in Los Angeles County, California, USA. Environ. Health Perspect..

[39] CDC. (2005). Hurricanes—special population. Effects on pregnant women—carbon monoxide. Retrieved from: www.cdc.gov/ncbddd/hurricanes/environmental.htm (Accessed on June 19, 2021).

[40] Abbey M, Adebari OO, Green KI, Chinko BC (2022). Carbon monoxide (CO) pollution in the Niger Delta area of Nigeria and its impact on foeto-maternal health. Sch. Int. J. Obstet. Gynecol..

[41] European Environment Agency (2022). Emissions and energy use in large combustion plants in Europe, https://www.eea.europa.eu/ims/emissions-and-energy-use-in (Accessed 18 February 2022)

[42] Hou H, Wang D, Zou XP, Yang ZH, Li TC, Chen YQ (2014). Does ambient air pollutants increase the risk of fetal loss? A case–control study. Arch. Gynecol. Obstet..

[43] IQAir Air quality in Nigeria (2022) https://www.iqair.com/nigeria

[44] Emetere ME, Akinyemi ML, Oladimeji TE (2016). Statistical Examination of The Aerosols Loading Over Kano-Nigeria: The Satellite Observation Analysis. Scientific Rev. Eng. Environ. Sci..

[45] Emetere ME (2016). Statistical examination of the aerosols loading over Mubi-Nigeria: The satellite observation analysis. Geographica Panonica.

[46] Brown JE, Alfonso B, Avila R, Beresford NA, Copplestone D, Hosseini A (2016). A new version of the ERICA tool to facilitate impact assessments of radioactivity on wild plants and animals. J. Environ. Radioact..

[47] Inskip MJ, Franklin CA, Baccanale CL, Manton WI, O’Flaherty EJ, Edwards CM (1996). Measurement of the flux of lead from bone to blood in a nonhuman primate (Macaca fascicularis) by sequential administration of stable lead isotopes. Fundam. Appl. Toxicol..

[48] Ehrmann W, Schmiedl G, Beuscher S, Krüger S (2017). Intensity of African humid periods estimated from saharan dust fluxes. PLoS ONE.

[49] Minka NS, Ayo JO (2014). Influence of cold–dry (harmattan) season on colonic temperature and the development of pulmonary hypertension in broiler chickens, and the modulating effect of ascorbic acid. Open Access Anim. Physiol..

[50] Aweda FO, Falaiye OA, Babatunde GJ (2017). Elemental concentration of Harmattan dust sample in Iwo and Oyo Town, South West Nigeria. J. Appl. Sci. Environ. Manag..

[51] Jimoh WLO (2012). chemical composition and mineralogy of Harmattan dust from Kano and Zaria Cities in Northern Nigeria. Res. J. Environ. Earth Sci..

[52] Okuo JM, Ndiokwere CL (2006). Elemental concentrations of total suspend particulate matter in relation to air pollution in the Niger Delta of Nigeria: A case study of Warri. Trends Appl. Sci. Res..

[53] Oluyemi EO, Asubiojo OI (2001). Ambient air particulate matter in Lagos, Nigeria: A study using receptor modelling with X-ray fluorescence analysis. Bullet. Chem. Soc. Ethiop..

[54] Ogundele TL, Olasinde RT, Owoade OK, Olise FS (2018). Composition and source identification of chemical species in dust from selected indoor environments in Ile-Ife Nigeria. Earth Syst. Environ..

[55] MNCH2 Maternal deaths in Kano (2020) https://www.mnch2.com/kano-state/

[56] Abdulkadir M, Rainis R (2020). Trends and causes of maternal mortality at the general hospitals in Jigawa north-west senatorial district. J. Crit. Rev..

[57] Akinyemi ML, Emetere ME, Akinwumi SA (2016). Dynamics of wind strength and wind direction on air pollution dispersion. Asian J. Appl. Sci..

[58] Adegoke AA, Campbell M, Ogundeji MO, Lawoyin TO, Thomson AM (2013). Community study of maternal mortality in South West Nigeria: How applicable is the sisterhood method. Matern. Child Health J..

[59] Emetere M. E. Environmental Modeling Using Satellite Imaging and Dataset Re-processing, Springer (2019). (ISBN 978–3–030–13404–4)

[60] Abubakar IS, Zoakah AI, Daru HS, Pam IC (2003). Estimating maternal mortality rate using sisterhood methods in plateau state Nigeria. Highland Med. Res. J..

[61] WHO Nigeria: WHO statistical profile (2013) https://www.who.int/gho/countries/nga.pdf?ua=1

[62] Grant M.P. Evaluation of Silica Exposures During Dowel Drilling (2020) https://www.cdc.gov/niosh/hhe/reports/pdfs/2019-0178-3368.pdf?s_cid=102015-HETAB-RSS-001

[63] HSE The HSE guidance on a range of dusts and sectors (2006) www.hse.gov.uk

[64] Iñiguez C, Esplugues A, Sunyer J (2016). Prenatal exposure to NO_2_ and ultrasound measures of fetal growth in the Spanish INMA cohort. Environ. Health Perspect..

[65] Yana Richens Urinary catheterisation: Indications and complications (2022) https://www.britishjournalofmidwifery.com/content/other/urinary-catheterisation-indications-and-complications

[66] APKLAS MESOTHELIOMA Mesothelioma Left Lung Icd 10 (2021) https://www.apklas.com/mesothelioma-left-lung-icd-10/

[67] Zaynab Muhammed Chellube, Joseph Clement Akan* and Emmanuel Mshelia Assessment of some metals in roadside dust from Damaturu, Yobe State, Nigeria. CSN Annual International Conference, Workshop and Exhibition, Article Number: AD14654 (2018).

[68] Eckerman K, Harrison J, Menzel H-G, Clement CH (2012). Compendium of dose coefficients based on ICRP publication 60. Ann. ICRP.

[69] Adewumi AJ (2022). Heavy metals in soils and road dust in Akure City, Southwest Nigeria: Pollution, sources, and ecological and health risks. Expo Health.

[70] Alhassan AJ, Sule MS, Atiku MK, Wudil AM, Dangambo MA, Mashi JA, Ibrahim NA (2012). Study of correlation between heavy metal concentration, street dust and level of traffic in major roads of Kano (Northwest) Nigeria Metropolis, Nigeria. Niger. J. Basic Appl. Sci..

[71] Adekola FA, Dosumu OO (2001). Heavy metal determination in household dusts from Ilorin City, Nigeria. Niger. Soc. Exp. Biol. J..

[72] Mafuyai GM, Eneji IS, Sha’Ato R (2014). Concentration of heavy metals in respirable dust in Jos Metropolitan Area, Nigeria. Open J. Air Pollut..

[73] Ademola AK (2021). Radiological risks from natural radionuclides in surface soil of Agbara industrial area, Ogun State Nigeria. J. Appl. Sci. Environ. Manag..

[74] Sombo T, Entonu S, Igbawua T, Shivil J (2021). Radionuclide content of aerosols within the lower atmosphere of major towns in Plateau State, North Central Nigeria. Niger. Ann. Pure Appl. Sci..

[75] Nimyel SH, Namadi MM (2020). Assessment of the level of heavy metal concentration in the street dust in some selected locations in Zaria Metropolis, Kaduna State, Nigeria. Fudma J. Sci..

